# The Treatment of Cancer

**Published:** 1859-05

**Authors:** 


*The Treatment of Cancer.—Cancer-Quacks.*

An unusual amount of attention has been given of late to certain methods of treating cancerous disease, and there now seems to be an evident leaning in many quarters, toward the employment of escharotics with more frequency than for some time past. While the circumstances of each particular case must modify the procedure of the Surgeon, it seems well to use the approved caustics in those instances, and in those particular species of cancerous growth, where the size and form, as well as the nature of the morbid product, admit of the practice. Locality must always have a vast deal of influence in deciding the practitioner as to the peculiar measure he should adopt. He would be more loth, we conclude, to apply escharotics to facial cancer, of whatever description, than he would to other regions of the body. Cancerous growths about the mouth, for instance, are far more properly treated by direct excision, than by caustics. In ordinary epithelial cancer of the lip, the knife is usually remarkably successful.

We are partly induced to refer to this subject—which is always one of great interest to the surgeon, and any improvements in which are of such vital importance to the sufferers from this terrible disease—on account of having had several instances brought under our personal observation, where the direst and entirely irremediable mischief has been done to the patient by medical pretenders. These harpies of society never have it in their power to do more downright injury to their unhappy victims, than when a veritable case of cancer is entrusted to their ignorant management. We say when a *genuine case is entrusted* to them—for a great majority of the so-called cancer cases which quacks report as being under their care are either pure fabrications, or else not cancer at all. Every ordinary glandular or other benign tumor which they secure for treatment is trumpeted as cancer—"an enormous cancer"—"a large rose cancer with extensive roots," &c., &c. Of this wholesale lying we have occasionally had proof—and in one instance, not long ago, when the merest tyro in medicine and surgery would have known better, a simple fatty tumor was pronounced, by a high-priest of "Indianopathy" in this city, to be a dangerous cancerous growth! And yet this class of piratical practitioners are able to parade a long list of names of "Reverend" gentlemen at the foot of their lengthy advertisements—interspersed, now and then, with the names of fabricators of surgical appliances; and in one instance, of one such who has been more's the pity we think—liberally patronized by our profession.

But matters are far worse when such imposters are bold and bad enough to undertake the management of a case of *bona fide* cancer; and it is truly wonderful how any one with the amount of evidence which every honest medical man could easily lay before him, can be hardy enough to run into such peril as to trust them. The more brazen-faced the quackery, however, the more eagerly it seems to be embraced; and not only are the chief agents in the knavery to be condemned, but also the editors of all such newspapers, as, for filthy lucre's sake, give up column after column to their mendacious advertisements. We are sorry to say that we have such in our city—to their everlasting disgrace be it spoken.

A most painful instance of mal-practice—after the "Indian Doctor" method—lately came to our knowledge. The case is such, as, in the opinion of good legal judges, would render the parties who practiced upon the patient, amenable to the heaviest damages, did the injured person or his friends choose to move in the matter—and this may yet be done. A few additional similar cases would, if exposed, be sufficient, without legal process, to break up the iniquitous dens whence these wrongs emanate.

The instance to which we have just referred, was originally one of facial cancer, of small extent, and perfectly amenable to treatment by excision. This measure was advised by a skillful surgeon of this city; but the deluded patient, being over-persuaded by some of his so-termed *friends*, placed himself under an "Indian Doctor," established near the centre of the city; and in consequence of his tampering with the disease, has gradually been reduced to an incurable condition. Nearly one half of the face being destroyed, in this patient, it is easy to conceive of the deplorable state he is in, and the vain regrets he and his friends must feel in view of the cause thereof. There was every reason to presume that a definitive cure would have been the result, had the advice of the surgeon referred to been been followed.

Not to add, at present, to the catalogue of horrors for which cancer quacks are answerable—by the aid of human weakness and credulity—will, for a moment, again refer to the use of caustics.

The chloride of zinc enjoys a well deserved reputation for the purpose of enucleating cancerous or epithelial growths. This is especially mentioned, of late (March 12, 1859), by a correspondent of the *Lancet*—Mr. Wordsworth, Assistant Surgeon to the London Hospital, and to the Royal London Ophthalmic Hospital. In one part of his communication, he remarks :—"it will be said that chloride of zinc will answer almost every purpose for which escharotics are employed ;" and he believes that a sufficient trial of it "will lead others to the same conclusion which he has formed of its great superiority over all other potential cauteries. In another place, he says, comparing the actual and potential cautery :—

"In many instances, the application of the 'actual cautery' is not so convenient as that of the 'potential' ones. Under such circumstances, I believe none can be more effectively and certainly used than the chloride of zinc. It can be managed with the greatest precision, and so regulated as to produce a slough of the thickness of brown paper, or, if desired, of half an inch in depth. Its use is adapted for those cases in which it is desired to arrest a morbid action ; as, for instance, the varied forms of phagedæna, or other specific ulcerations, in which a *superficial* slough only is required ; or for the enucleation of cancerous or epithelial growths, in which the surface is ulcerated, and from their extent, or some other condition, the knife is inadmissible. Its effect is far more extensive than that of the acids, and, with a little care can be easily circumscribed. Several years ago, the writer was induced to combine it with oxide of zinc as a convenient vehicle for diluting, and applying it to a sore.

"Experience has amply proved the advantages which this combination possesses over those commonly recommended, such as flour or plaster-of-Paris; for while with tne oxide it remains as a *powder* easy of application, so as to produce a certain and uniform result, with the latter means it forms a *paste* of some consistence, and becomes much less manageable. The oxide also possesses the peculiar quality of protecting the chloride from deliquescence, and so allows it to be kept for a considerable (if not indefinite) period, and the mixture is always ready for use."

In perusing Mr. Pemberton's late work on melanotic cancer, we observe that he is strongly in favor of the use of caustic in that particular form of the disease; indeed he terminates his volume with a recommendation to employ escharotics mainly, or at least to bring them to the aid of the knife, believing the result of such a practice to be more encouraging than those secured by pure excision. The use of the cautery and of caustics is so greatly facilitated by anæsthetics, that they may often be advantageously employed, where in former times they would not have been deemed admissible—or where doubts might have been reasonably entertained whether their application could have been endured.—*Boston Medical & Surgical Journal.*

